# Interfacial Biomaterial–Dentin Bacterial Biofilm Proliferation and Viability Is Affected by the Material, Aging Media and Period

**DOI:** 10.3390/dj10030033

**Published:** 2022-02-24

**Authors:** Muna Q. Marashdeh, Celine Lévesque, Shimon Friedman, Cameron A. Stewart, Yoav Finer

**Affiliations:** 1 Faculty of Dentistry, University of Toronto, Toronto, ON M5G 1G6, Canada; muna.marashdeh@mail.utoronto.ca (M.Q.M.); celine.levesque@dentistry.utoronto.ca (C.L.); s.friedman@utoronto.ca (S.F.); cameron.stewart@mail.utoronto.ca (C.A.S.); 2 Institute of Biomedical Engineering, University of Toronto, Toronto, ON M5S 3E2, Canada

**Keywords:** biomaterial–dentin interface, biofilm proliferation, salivary enzymes, *Enterococcus faecalis*, bioceramic sealer, epoxy-resin sealer, resin composite, dentistry, interface

## Abstract

Biomaterial–dentin interfaces undergo degradation over time, allowing salivary, tissue fluid, and bacterial movement between the root filling or restoration and dentin. This study aims to investigate the effect of aging in simulated human salivary/bacterial/blood esterases (SHSE) on proliferation and viability of *Enterococcus faecalis* biofilm within the dentin interface with four materials used to fill/restore the endodontic space. Root canals of human anterior teeth were prepared and filled with gutta-percha and one of the following: self-cured resin composite (Bisfil^TM^ 2B, Bisco, Schaumburg, IL, USA) with either self-etch (SE) (EasyBond) or total-etch (TE) (Scotchbond^TM^, 3M, Saint Paul, MN, USA) methacrylate-based adhesives, epoxy-resin sealer (AH Plus^®^, Dentsply Sirona, York, PA, USA), or bioceramic sealer (EndoSequence^®^ BC Sealer™, Brasseler USA, Savannah, GA, USA). Specimens were aged in SHSE or phosphate-buffered saline (PBS) for up to 360 days, followed by cultivation of steady-state *E. faecalis* biofilm. Depth and viability of interfacial bacterial biofilm proliferation were assessed by confocal laser scanning microscopy and live/dead staining. Data were analyzed using three-way ANOVA and Scheffe’s post hoc analyses. Initial depths of biofilm proliferation were similar among material groups (*p* > 0.05). All groups showed significantly deeper biofilm proliferation with increased aging period (*p* < 0.05). SHSE aging increased interfacial biofilm depth for TE, SE and BC (*p* < 0.05) but not AH. For unaged interfaces, BC exhibited the lowest ratio of live bacteria, followed by AH, TE, and SE (*p* < 0.05). Interfacial bacterial biofilm proliferation and viability were dependent on the biomaterial, aging media, and period.

## 1. Introduction

Bacterial biofilms are the primary cause of root canal infections. Microbes persist in the root canal system even after thorough chemomechanical preparation and can proliferate to levels similar to those before treatment [1]. *Enterococcus faecalis* is a microorganism commonly detected in endodontic infections [2]. The survival of *E. faecalis* after endodontic therapy has been attributed to its ability to form biofilm, degrade dentinal collagen and methacrylate resin, and to survive harsh conditions [2,3,4]. Root canal sealers, used for root filling along with a core material, fill the interface between the core material and canal walls to entomb residual bacteria and prevent bacterial ingress and biofilm proliferation. An ideal endodontic sealer should exhibit antimicrobial activity, an excellent seal when set, dimensional stability, tolerance to tissue fluids, and mechanical strength [5].

Epoxy-resin-based sealers, such as AH Plus (AH) are considered the “gold standard” due to favorable physical and biological properties and proven clinical performance; however, they are limited by low bond strengths to core material and diminished antimicrobial activity after setting [6]. New bioceramic (BC) sealer has been recently introduced as a contemporary hydraulic calcium-silicate-based cement that uses the water in dentin to initiate and complete its setting reaction and to form hydroxyapatite, resulting in a chemical bond to the dentinal canal wall [7]. Methacrylate resins were proposed as root canal sealers but their performance is inferior [8]; they are currently used mainly for post-cementation [9] and restoration [10]. Resin adhesive systems are intended to bond resin composites to tooth structure and improve the sealing of the root canal filling [8]. Two main adhesive systems are currently available: total-tech systems (TE), designed to remove the smear layer, and self-etch systems (SE), designed to modify the smear layer [11]. Both adhesive systems rely on the formation of a resin-infiltrated dentinal collagen network for bonding, known as the hybrid layer [12].

Biomaterial–dentin interfaces undergo degradation over time, allowing salivary, tissue fluid, and bacterial movement between the root filling or restoration and dentin [12,13,14]. Human salivary esterase (as measured in saliva or simulated with an enzyme mix in vitro), bacteria, and neutrophils affect the physical properties of this interface and accelerate the degradation process in a manner that is dependent on the chemical composition of the restorative materials used [3,14,15,16]. Degradation by-products affect the gene expression and protein synthesis of cariogenic bacteria, and potentially their virulence [14,17]. The compromised interface of dental restorations allows bacterial penetration, dentin degradation, and bond strength weakening [12,18], which in the endodontic context may lead to development of post-treatment apical periodontitis [19]. However, to the best of the authors’ knowledge, these interactions have not been assessed in the endodontic context. Therefore, existing and newly developed sealers and restorative materials used to restore the endodontic space should be assessed for their interfacial integrity and biostability via aging in degradative media relevant to intraoral conditions.

The aim of this study was to investigate the effect of aging endodontically treated teeth in simulated human salivary/bacterial/blood esterases (SHSE), for up to 360 days, on the proliferation and viability of *E. faecalis* biofilm within the interface between root dentin and four different types of materials used to fill/restore the endodontic space. *E. faecalis* may not be the only pathogen responsible for root canal infection, but it is a very well understood bacterial factor and remains a useful biomarker for bacterial penetration in the context of the goals of this study. The investigated materials are representative of methacrylate-resin composites with self-etch or total-etch bonding, epoxy-resin-based sealers, and hydraulic calcium-silicate sealers. We hypothesized that the interfacial biofilm proliferation and viability will be affected by the type of biomaterial, aging medium, and period, thus demonstrating the importance of simulating the biodegradative factors in the mouth when analyzing endodontic sealer performance.

## 2. Materials and Methods

### 2.1. Specimen Preparation

Power analysis was performed using results from a pilot study to determine sample size requirements for effect observation (α = 0.05, 1 − β = 0.8, G*Power Version 3.1.9.2 Software, Heinrich-Heine-Universität Düsseldorf, Dusseldorf, Germany). Effect size minimum was set to one unit (1 µm or 1 point of live/dead ratio change). One Way ANOVA test would require a sample size of 2 teeth/group/time point to achieve 80% statistical power. In the present study 3 teeth/group/time point were used.

Human anterior caries-free teeth were collected and kept stored at −20 °C in distilled water (University of Toronto ethical approval # 28214) until ready for use [3,12,20]. Teeth were inspected for cracks under an operating microscope and only crack-free teeth were selected. Tooth crowns were dissected, and subsequent endodontic treatment procedures performed under aseptic conditions. Root canals were gradually enlarged from 0.9–1.5 mm with parallel drills (ParaPost^®^, Coltene, Cuyahoga Falls, OH, USA), and the roots were inspected again for cracks. Prepared roots were sterilized by autoclave, which has been demonstrated not to affect dentin properties [21]. Canals were irrigated with 5 mL of 5.25% NaOCl (Millipore Sigma, Burlington, MA, USA), 5 mL of 17% EDTA (Millipore Sigma, Burlington, MA, USA), a final flush of 10 mL of sterile distilled water and dried with sterile paper points. Roots were randomly assigned to one of four experimental groups: SE, TE, AH and BC (Table 1). Roots were filled with gutta-percha points and the group-specific biomaterial as sealer, and stored for 72 h in a 100% humidity environment at 37 °C.

A 3 mm root segment was obtained from the coronal part of each root using a slow speed, water-cooled rotary diamond saw. The apical and coronal surfaces of these root segment specimens were polished with 600–1200-grit silicon carbide grinding papers [16]. All exposed coronal dentin adjacent to root fillings and cementum was coated with clear varnish to prevent bacterial penetration into the sealer–dentin interface through cut dentinal tubules.

### 2.2. Specimen Aging

Specimens (*n* = 3/material/time point) were either unaged (control), or aged in either SHSE, or phosphate-buffered saline (PBS) at 37 °C, pH 7.0 for 30, 180, and 360 days. SHSE was prepared by dissolving cholesterol esterase (CE) and pseudocholine esterase (PCE) in PBS. Incubation solutions were replenished throughout the aging period to keep the esterase activities at levels corresponding to activities present in human saliva, as previously described [18], maintaining 16 U/mL and 0.01 U/mL for CE and PCE activity, respectively.

### 2.3. Biofilm Cultivation

Following the assigned aging period, specimens from each incubation period were suspended in a Chemostat Based Biofilm Fermenter (CBBF) to cultivate steady-state biofilms of *E. faecalis* ATCC 29212 for 3 days in Brain heart infusion (BHI)(pH = 7.0, 37 °C). An overnight culture of *E. faecalis* ATCC 29212 in BHI was used to inoculate the CBBF, before pumping fresh medium (1/2 BHI supplemented with 20% glucose *w*/*v* and 40 mM phosphate citrate buffer) into the vessel at a flow rate = 1.6 mL/min, mimicking human salivary dilution rate [12,20,22]. Controls of the following were prepared to provide Confocal Laser Scanning Microscope (CLSM) baseline readings: (1) no aging, incubation in the CBBF without bacterial cells, and staining, (2) no aging, incubation in the CBBF with inoculation, and staining or (3) no aging, no incubation in the CBBF, and staining.

### 2.4. Outcome Assessment

At the end of their respective aging period, specimens were removed aseptically from the CBBF, gently rinsed with distilled water, and stained using LIVE/DEAD^®^ stain (BacLight™ Bacterial Viability Kit, Invitrogen, Waltham, MA, USA). Stained specimens were assessed individually for marginal interface morphology, and bacterial biofilm proliferation, penetration and viability using CLSM (Zeiss LSM710 Two-photon and confocal microscope, Carl Zeiss Canada Ltd., Toronto, ON, Canada). The biomaterial–dentin interface was identified, and three standardized regions of interest scanned using a 20X (water-immersion)/NA 1.0 objective lens for a total of 9 scanned regions per experimental group. Z-stack sequential images were collected at 1 μm intervals between images. CLSM Z-stacks were processed by IMARIS (Bitplane AG, Zürich, Switzerland) [23].

### 2.5. Statistical Analysis

After testing for normal distribution (Shapiro–Wilk test) and homoscedasticity (residual plots), three-way ANOVA and Scheffe’s post hoc analyses (*p* < 0.05) were used to determine the effect of biomaterial type, aging media, and time, on the depth of bacterial proliferation identified within the biomaterial–dentin interface and the proportion of live bacteria.

## 3. Results

### 3.1. Bacterial Biofilm Proliferation Depth

**Figure 1:**
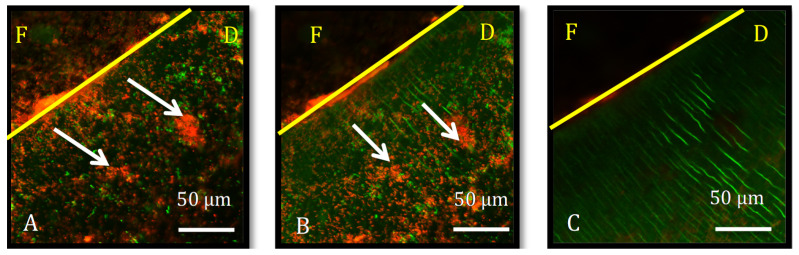
Representative confocal Z-stack images processed by IMARIS, of a specimen from group AH, aged for 30 days in PBS. The yellow line marks the interface between root canal filling (F), and dentin (D), at depth 0 μm (**A**) and 5 μm (**B**). White arrows show bacterial biofilm. At 13 μm (**C**) there were no visible biofilms. Live/Dead kit, green cells are live while red cells are dead.

Bacterial biofilm proliferation into the biomaterial–dentin interface was observed in all specimens and is demonstrated in an example from group AH (Figure 1). the 30-day BC group could not be assessed due to material expansion that blurred the interface.

**Figure 2:**
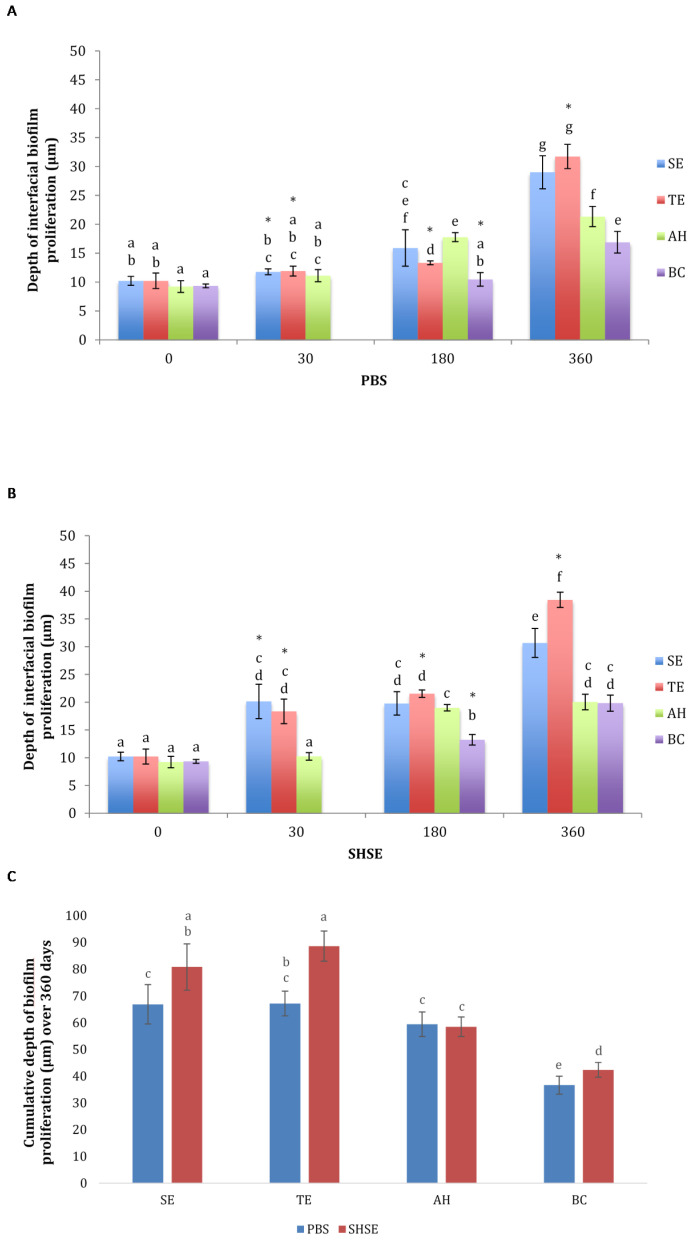
Interfacial biofilm proliferation depth (μm) for the test groups (SE, self-etch adhesive; TE, total-etch adhesive; AH, epoxy-resin sealer; BC, bioceramic sealer) before and after aging for 30, 180 and 360 days in either (**A**) PBS or (**B**) SHSE. (**C**) Maximum depth of biofilm proliferation (μm) for all aging periods in either PBS or SHSE for each test material group. Data are shown as mean ± SD. BC had the lowest bacterial penetration (**C**). Different letters represent statistically significant differences between materials at different aging periods within the same aging medium. * Indicates significant difference between PBS and SHSE for the same material at same aging period.

Biofilm proliferation depths for different groups, aging periods, and media are depicted in Figure 2. Before aging (control), interfacial bacterial proliferation did not differ significantly among the four groups (*p* > 0.05). After 360 days of aging, all four groups showed significantly deeper biofilm proliferation, regardless of aging in PBS or SHSE (*p* < 0.05). Overall, the deepest biofilm proliferation was observed in TE and SE groups, followed by AH and BC groups, with variation in significance depending on aging in PBS or SHSE.

SE and TE groups showed significant increase in biofilm proliferation depth after 30 days of aging in SHSE (*p* < 0.001), and after 180 days of aging in PBS (*p* < 0.05). AH exhibited a significant increase in biofilm proliferation depth after 180 days of aging in both SHSE and PBS (*p* < 0.05).

The cumulative biofilm proliferation depth of all aging intervals was higher for aging in SHSE than for aging in PBS for SE, TE, and BC groups. Incubation media did not significantly affect depth of bacterial penetration in AH specimens.

### 3.2. Proportion of Live Bacteria

**Figure 3:**
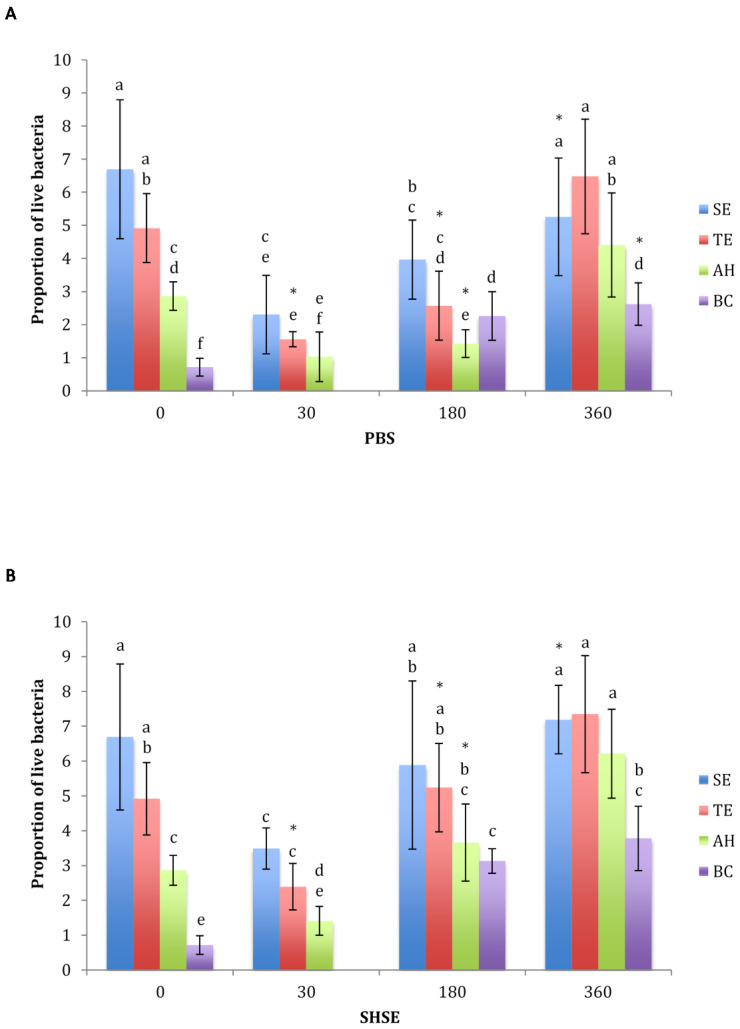
Proportion of live bacteria in interfacial biofilms for the test groups (SE, self-etch adhesive; TE, total-etch adhesive; AH, epoxy-resin sealer; BC, bioceramic sealer) before and after aging for 30, 180, or 360 days in (**A**) PBS or (**B**) SHSE. Data are shown as mean ± SD. * Indicates significant difference between PBS and SHSE of the same material at same time point.

Proportion of live bacteria for different materials, aging periods, and media, are depicted in Figure 3. Before aging, the proportion of live bacteria was significantly lower in BC than AH, TE and SE (*p* < 0.05). In all four groups, a significant decrease in proportions of live bacteria after 30 days of aging in both PBS and SHSE was followed by a significant increase after 180 days of aging in both media (*p* < 0.05). Aging in SHSE yielded significantly higher proportions of live bacterial for TE at 30 days and 180 days, AH at 180 days, SE at 360 days, and BC at 360 days (*p* < 0.05). After 360 days of aging in either PBS or SHSE, BC sealer showed the lowest proportion of live bacteria (*p* < 0.05).

## 4. Discussion

This in vitro investigation monitored the proliferation of *E. faecalis* biofilm between root dentin and four different types of materials used to fill/restore the endodontic space, as a marker of biomaterial–dentin interface integrity. The penetration of bacteria in the interface is facilitated by the separation between material and dentin and the formation of a void through which bacteria may travel, both indications of insufficient seal and/or interfacial breakdown. None of the tested materials provided a bacteria-tight initial seal of the interface. Increased biofilm proliferation was detected in specimens aged for different time periods, suggesting degradation of the sealer–dentin interface over time affects penetration. The depth of biofilm proliferation and viability, and thus the implied extent of interfacial degradation, depended on material type, aging medium, and period, and thus the initial hypothesis may be accepted.

Specimens were aged individually in sterile glass vials containing either PBS or SHSE for up to 360 days. Following the aging period, specimens were incubated in CBBF to allow for continuous controlled flow of BHI medium, simulating pathogenic oral conditions to cultivate *E. faecalis* biofilm [12,13,20], a facultative anaerobic coccus that was used as a biological marker to assess the interfacial stability and is a representative endodontic pathogen that is easily cultivable and therefore a suitable biomarker for endodontic studies. In this capacity *E. faecalis* appeared suitable given the present results and past research [3,4,11,12,15,16,18,20,24]. However, care should be taken to not interpret the data here as 1:1 with clinical results; a multispecies approach may be necessary to interpret the true pathological significance of increased bacterial penetration and proliferation due to material biodegradation. The rationale for using different SHSE or PBS aging periods before incubation with *E. faecalis* was based on the need to prevent interactions between the proteins in SHSE and the bacteria to isolate their true effect, and the practical limitations of long-term biofilm growth under in vitro conditions [12,20].

SHSE, used in this investigation to mimic salivary, blood, bacterial, and neutrophil esterase-like activity in the oral cavity, accelerated interfacial degradation of BC, TE and SE and increased the proportion of live bacteria within the sealer–dentin interface in all materials at different time points. SHSE is a mixture of CE and PCE that act synergistically with hydrolytic activity levels similar to those seen in situ in the oral cavity to increase biodegradation of methacrylate-resin dental composites and adhesives, decrease their bond integrity to dentin [18], increase biofilm proliferation within the adhesive–dentin interface [12], decrease microhardness, increase weight loss, and cause dimensional change. All of these factors may in turn affect the clinical performance of resin adhesives and bioceramic sealers [16]. The findings of this study highlight the importance of using SHSE to mimic intraoral enzymatic conditions when testing biomaterial intended for applications in endodontics.

CLSM is a non-destructive technique that enables examination of biofilms in situ without the limitations encountered by two-dimensional destructive and static methods, such as scanning electron microscopy [12,25]. CLSM is used to determine the true architecture of plaque and the location of selected bacteria within the biofilm [26]. However, the use of CSLM can be difficult with clinically obtained samples; confocal microscopy has limited depth of penetration and analysis requires parallel canal walls, while clinically canals are tapered. Furthermore, one of the sealers used (BC) is opaque, limiting the depth of analysis across all material groups in the present study.

Using a suite of fluorescent stains allows quantification of the cells within the biofilm and determination of viability [25], which may be used as an indication of the antimicrobial activity or cytotoxicity of the biomaterial [27]. In the current investigation, the stained bacteria were used to assess whether the conditions that formed within the biomaterial–dentin interface were favorable to the formation of live pathogenic biofilms.

The capacity of all test materials to seal the canal and prevent bacterial penetration, comparable before aging, deteriorated with exposure to both media. BC aged in PBS for 360 days was the least prone to interfacial bacterial proliferation of all test materials aged in both media. This might suggest a more stable material–dentin interface but may also be a product of the prolonged alkaline pH of the sealer and the release of bioactive glass [28]. Exposure of BC to SHSE was previously shown to reduce the material setting expansion and increase its solubility [16]. In the current study, the difficulties encountered with imaging the 30 day-aged BC specimens suggest initial expansion of the setting material that obstructed the CLSM field of view. Subsequent aging and possible material dissolution reduced visual obstruction and allowed for analyses via CLSM.

AH was previously shown to be more hydrolytically stable than other sealers [16]. The current study corroborated these findings by showing that AH was only minimally affected by exposure to SHSE, possibly due to the material hydrophobicity and lack of susceptible ester bonds, imparting this material with resistance to hydrolysis [29].

TE and SE methacrylate resin–dentin interfaces were the most susceptible to interfacial biofilm proliferation. SHSE incubation increased this susceptibility, most likely due to hydrolysis of the ester-links within these methacrylate-resin materials. In addition, these materials’ initial post-polymerization shrinkage may contribute to interfacial degradation, compromised seal, and interfacial biofilm proliferation noted in the current investigation [16]. Despite being more hydrophilic and, therefore, more susceptible hydrolytic degradation and reduction in mechanical properties [16], SE specimens exhibited less bacterial penetration than TE after 360 days aging in SHSE. This result agrees with those of Serkies et al. [18], which showed SE’s superior strength and preservation of interfacial integrity vs. TE after aging in SHSE. The increased performance of SE over TE could be due to the complex root canal geometry and difficulty of applying material therein [30]; SE does not require a separate etch and rinse step and thus reduces risk of user error in this environment. Furthermore, SE’s inherent differences in chemical composition could contribute to its greater resistance to enzymatic attack [24]. Previous work has suggested that endogenous matrix metalloproteinases in dentin may be activated by the phosphoric acid-etch step of TE systems; however, our own experience is that this activity is limited and significantly less than *E. faecalis’s* inherent collagenolytic activity, and thus is unlikely to be a differentiating factor for TE performance [4,18]. Inferior performance of restorative adhesives as root canal sealers in the current study is in agreement with previous data [8]; however, their inclusion in this study is useful as a positive control of interface degradation susceptibility, and the present results help clarify the source of their inferior endodontic sealing performance.

Increasing proportions of live bacteria in some of the SHSE aged groups could be explained by possible interactions between the constituent proteins of SHSE and the bacteria, as well as the increase in interfacial gap produce by the degradative activity of SHSE on the material over time [12,31]. BC showed the lowest proportions of live bacteria of all groups, suggesting antimicrobial activity, possibly due to the material’s alkaline pH. AH and BC antimicrobial activity reduced over time, corroborating previous studies [32,33]. SE, TE and AH were previously reported to have initial antimicrobial activity after setting [13]. However, all material specimens exhibited lower proportions of live bacteria after 30-days of pre-incubation than at 0-days. Although the release of cytotoxic compounds from sealers, and therefore any antimicrobial effect, is expected to be highest immediately post-placement, these results suggest otherwise. There may be a delay in cytotoxic effect caused by the slow initial diffusion of cytotoxic compounds from sealers, the delay in generation of biodegradation by-products by enzymatic breakdown (in SHSE groups), the slow diffusion of compounds from the interface to the oral cavity, and potentially a resultant accumulation of these compounds in the interface over time. The interfacial concentration of these cytotoxic compounds may reach antimicrobial levels closer to the 30-day timepoint in the current study. Sometime after the 30-day timepoint and before the 180-day timepoint, the balance between diffusion into and out of the interface may shift, leading to a decline in cytotoxic compound concentration in the interface and a decline in antimicrobial effect. This observation suggests that testing for interfacial or eluent cytotoxicity over a length of time is more relevant than testing the immediate cytotoxicity of the sealer alone when assessing the performance of endodontic biomaterial, as latent antimicrobial and cytotoxic effects may only be seen after the accumulation of relevant sealer eluents.

Although the current study was successful in demonstrating the importance of biomaterial-biodegradative factors in endodontics, future studies should further leverage this direction to assess the disease impacts of these interactions. The current study is limited by the monospecies nature of the biofilm used, the static thermomechanical nature of the incubation, and the simulated nature of enzymatic degradation. Next steps would almost certainly include using multispecies biofilms or in situ study which may overcome these two factors through “incubation” of an appliance-bound specimen in a human mouth. In addition, non-destructive techniques to assess biofilm penetration or damage to host tissues and biomaterial should continue to be leveraged to overcome the limitations of CLSM analysis of clinical samples. Full clinical study remains an elusive goal, as testing chemical degradation and interfacial biofilm properties of sealers in their clinical indication without destroying the treatment would be exceedingly challenging.

This study also evaluated endodontic treatment only along a confined set of parameters. Although we believe that the interactions between various material chemistries and biodegradative factors is important to long-term performance, testing these interactions in the presence of other treatment improvements such as antimicrobials, remineralizing agents, or biofilm-modulating factors will be critical in the continued development of new endodontic techniques and materials. Likewise, these new treatment options should be tested in the presence of biodegradative factors to gain a more complete understanding of their potential.

## 5. Conclusions

The results of the current study clearly demonstrate that the interaction of biodegradative media and root canal sealers has a significant impact on pathogen biomarkers, and thus be more seriously considered going forward. Exposure to biodegradative media mimicking the hydrolytic capacity of saliva, blood, bacteria, and cells of the immune system impacted the interfacial biofilm proliferation, depth, and proportion of live bacteria for test materials and highlighted the importance of using aging media with enzymatic activities relevant to the oral cavity for the assessment of biomaterial used in endodontics. Going forward, designers of new materials for endodontic materials should strive for hydrolytic stability even in the absence of bacteria, and clinicians must remain cognizant of not just product handling and immediate sealing, but how these materials may interact with the patient and degrade over time.

## Figures and Tables

**Table 1:** Description of the materials used as sealers in the experimental groups.

Group	Description
SE	Resin composite (Bisfil^TM^ 2B, Bisco, Schaumburg, IL, USA) bonded to root dentin using self-etch adhesive (Adper^TM^ Easy Bond, 3M, Saint Paul, MN, USA).
TE	Bisfil^TM^ 2B bonded to root dentin using total-etch adhesive (Scotchbond^TM^, 3M, Saint Paul, MN, USA)
AH	epoxy-resin-based sealer (AH Plus^®^, Dentsply Sirona, York, PA, USA).
BC	Bioceramic sealer (EndoSequence BC Sealer, Brasseler USA, Savannah, GA, USA)
